# The prominent role of the cerebellum in the learning, origin and advancement of culture

**DOI:** 10.1186/s40673-016-0049-z

**Published:** 2016-05-05

**Authors:** Larry Vandervert

**Affiliations:** American Nonlinear Systems, Spokane, WA USA

**Keywords:** Cerebellum, Creativity, Culture, Origin of culture, Language evolution, Sequence detection hypothesis, Stone tool evolution, Excessive television viewing, Thought dysmetria, Working memory

## Abstract

**Background:**

Vandervert described how, in collaboration with the cerebral cortex, unconscious learning of cerebellar internal models leads to enhanced executive control in working memory in expert music performance and in scientific discovery. Following Vandervert’s arguments, it is proposed that since music performance and scientific discovery, two pillars of cultural learning and advancement, are learned through in cerebellar internal models, it is reasonable that additional if not all components of culture may be learned in the same way. Within this perspective strong evidence is presented that argues that the learning, maintenance, and advancement of culture are accomplished primarily by recently-evolved (the last million or so years) motor/cognitive functions of the cerebellum and not primarily by the cerebral cortex as previously assumed. It is suggested that the unconscious cerebellar mechanism behind the origin and learning of culture greatly expands Ito’s conception of the cerebellum as “a brain for an implicit self.”

**Results:**

Through the mechanism of predictive sequence detection in cerebellar internal models related to the body, other persons, or the environment, it is shown how *individuals* can unconsciously learn the elements of culture and yet, at the same time, be in social sync with other members of culture. Further, this predictive, cerebellar mechanism of socialization toward the norms of culture is hypothesized to be diminished among children who experience excessive television viewing, which results in lower grades, poor socialization, and diminished executive control.

**Conclusion:**

It is concluded that the essential components of culture are learned and sustained not by the cerebral cortex alone as many traditionally believe, but are learned through repetitious improvements in prediction and control by internal models in the cerebellum. From this perspective, the following new explanations of culture are discussed: (1) how culture can be learned unconsciously but yet be socially in sync with others, (2) how the recent evolutionary expansion of the cerebellum was involved in the co-evolution of earliest stone tools and language—leading to the cerebellum-driven origin of culture, (3) how cerebellar internal models are blended to produce the creative, forward advances in culture, (4) how the blending of cerebellar internal models led to human, multi-component, infinitely partitionable and communicable working memory, (5) how excessive television viewing may represent a cultural shift that diminishes the observational learning of internal models of the behavior of others and thus may result in a mild, parallel version of Schmahmann’s cerebellar cognitive affective syndrome.

Elsewhere I proposed how the learning of cerebellar internal models during music training enhances executive processes in working memory and thereby can lead to scientific discovery and therapeutic efficacy [1, 2]. In brief, the following three-part unconscious cerebrocerebellar mechanism was proposed to be behind the production of high-level music performance and scientific contributions. *First*, through the detection of sequences in repetitive patterns during problem solving, the cerebellum unconsciously learns error-driven *internal models* of all behavioral, cognitive and affective processes that subsequently contribute to goal attainment in *working memor*y, and it uses these internal models to *adaptively* optimize the unconscious prediction and anticipation of similar future environmental events. This first mechanism is based directly on Akshoomoff, Courchesne and Townsend’s [3] and Leggio and Molinari’s [4] mutually supportive conclusions on the predictive, forward modeling role of the cerebellum in both movement and cognitive control processes. These researchers’ mutually (and independently) proposed relationship between cerebellar sequence detection and the learning of cerebellar internal models is clearly laid out in Leggio and Molinari’s [4] cerebellar sequence detection hypothesis:

According to this hypothesis, the cerebellum detects and simulates repetitive patterns of temporally or spatially structured events, regardless of whether they constitute sensory consequences of one’s actions in motor planning, expected sensory stimuli in perceptual prediction, or inferences of higher-order processes (e.g., cognitive elaboration or social cognition). The simulation allows *internal models* [italics added] to be created that can be used to make predictions about future events that involve any component, such as the body, other persons, and the environment. (p. 36)

Cerebellar internal models are learned as a neuronal circuits for the “forward-predictive” manipulation (control) of what in the cerebellum literature is referred to as a “controlled object.” As Leggio and Molinari point out in the above quote, such controlled objects include the body (for example, in using the hands, legs, arms), other persons (for example, in “controlling” the behavior of others in communicating, teaching, negotiating and so forth), and the environment (for example, everything from using stone tools to playing the piano (and assimilating the musical piece) to accessing information from iPhones). Thus, with repetitious practice, forward-predictive internal models in the cerebellum permit the unconscious manipulation of the forgoing controlled objects toward the achievement of goals. We will return to this learning of predictive cerebellar internal models in relation to socialization toward the norms of culture^1^ in more detail later in this article.

*Second*, within the framework of the forgoing cerebellar sequence detection and prediction process, unconscious cerebellar forward-predicting internal models are adaptively *blended* [5] in new prediction-optimizing ways during all problem solving, for example, in the culture components, music and science [1, 2]. *Third*, when the resulting unconsciously learned new blends of forward-predicting internal models are sent to consciousness in working memory, they are often experienced as sudden insight or intuition [1, 2]. These new blends of forward-predicting internal models may both advance the individual’s learning of the task at hand and contribute newly expanded knowledge in the form of innovation and creative discovery, for example, in music and science. This overall three-part *cerebro-cerebellar* mechanism of innovative and creative advancement may be summarized in the phraseology Leggio and Molinari [4] so aptly suggested in the title of their above-quoted article on cerebellar sequence detection, namely, “*Cerebellar Sequencing: a Trick for Predicting the Future*.”

## Purpose

If predictive sequence detection and blending in the cerebellum’s internal models indeed play a foundational/integral role in the above unconscious, step-by-step cerebrocerebellar advances in scientific discovery and expert musical performance, both highly sequence- or rule-based forms of cultural knowledge and technology, it then reasonably follows that cerebellar internal models may likewise play a foundational role as the driving mechanism behind many additional if not all aspects of culture (see Endnotes). Within this perspective, the purpose of this article is to propose how unconscious, forward-predictive internal models learned in the cerebellum may have played the dominant role both in i*nitiating* the first moments of the evolution of culture and in its further *elaboration* and advancement during the subsequent approximately 190,000 years of prehistoric and historic development (for example, Powell, Shennan & Thomas [6]). Specifically, it is argued that (1) Only the human cerebellum has evolved the new specialized cognitive functions in the last million years by which to unconsciously learn and refine the sharable, common skills, bodies of knowledge, beliefs, and language that through constant error-correction (Ito, [7, 8]); Leggio and Molinari, [4]; Leiner, Leiner, & Dow, [9, 10]) come to comprise culture (Vandervert, [1, 11]), and (2) to *share* this common, yet unconsciously learned culture, the skills, knowledges, and affective basis of culture can only consist of the learning of equally common *context-independent*^2^ cerebellar internal models among communicating humans as described by Doya [12], Imamizu and Kawato [13], Moberget, Gullesen, Andersson et al. [14] and Wolpert, Doya and Kawato [15]. Following directly in the vein of these latter three researches, it is proposed that this sharing of culture is accomplished through the learning of internal models of other persons as controlled objects (see the earlier Leggio & Molinari [4] quote and discussion). The tremendous, silent computational power of the human cerebellum and its vast neural connective relationship with the cerebral cortex will be described below.

In solid, preliminary support of these two arguments, Van Overwalle and Mariën [16] concluded that the cerebellum learns internal models for “social cognition” that are constantly error-corrected and sent to the cerebral cortex for the moment-to-moment, predictive “fluent and automatic social interaction” (p. 16). Of course, without *social cognition*, socialization toward enculturation could not occur. Therefore, Van Overwalle and Marien's conclusion comports with both Doya’s [12] description of the cerebellar modeling of speakers and listeners (including their nonverbal communication) as mutual control objects and with the proposal presented in this article that culture is adaptively driven by the learning of just such cerebellar internal models during socialization (see Endnotes). It is proposed that examples of this socialization toward the norms of culture can be seen in the repetitive, adaptive learning involved in the acquisition of culture-specific constellations of music, science, art, religion, and family practices for a particular culture; they are learned in cerebellar internal models in the same way as described for music and science by Vandervert [1], that is, in accordance with Leggio and Molinari’s [4] predictive scenario outlined in their above quote.

This new neuroanthropological perspective leads to the following, perhaps bold, assertion: While the large human cerebral cortex may have been the overwhelmingly dominant organ of selective advantage in the highly context-dependent world of natural selection that existed before the advent of culture, with the advantageous evolutionary enlargement of the cognitive regions of the cerebellum and their tool-making and communications-related skills (Vandervert, in press-a), *the cerebellum increasingly became the main driver behind the evolutionary advent of culture and its continued advancing development*. This somewhat speculative new perspective extends Ito’s [8] overall point in his book, “The Cerebellum: a brain for an implicit self.” In his book, Ito explains that the meaning of “an implicit self” derives from the fact that the cerebellum performs its contributions unconsciously. And that, even though learning to accomplish complex cognitive/motor tasks is performed within conscious awareness, when these tasks are refined through practice this awareness is taken over by unconscious cerebellar internal models. Ito specifically described what he meant by “implicit” as follows:

When we think about some topic repeatedly, the thought becomes more and more implicit; that is, it requires less and less conscious effort, as in intuition. This suggests that the cerebellum aids the self in both movement and thought, but covertly, by use of its internal models. ([8], pp. viii-ix)

In this article, this “implicit” self is unconsciously learned in direct relation to internal models of other persons as cerebellar controlled objects in social communication, including nonverbal communication (a la Doya [12], Imamizu and Kawato [13], and Wolpert, Doya and Kawato [15]), and thereby can be extended to the likewise “implicit” or unconscious origin, subsequent elaboration, and forward advance of culture.

Finally, implications of these arguments are followed into research evidence on the effects of modern, information technology-augmented culture and excessive television viewing among children. Here, it is hypothesized that since modern electronic devices (iPads, iPhones and television particularly) remove much of the burden of repetitious observational learning of cerebellar internal models of other persons as controlled objects (Doya [12]), cultural information is to a lesser degree being unconsciously learned. This lessening of a conscious/unconscious capacity to be in sync with cultural norms regarding attentional control, belief, and compliance may be seen in deficits in childhood education and psychological well-being which parallel those described in Schmahmann’s [17–19] dysmetria of thought.

## Traditional neuroscience approaches to the evolution of culture

The definition of culture used here refers to the shared beliefs and ways of doing things among the members of a particular group of people which are learned through socialization (see Endnotes). Others have proposed evolutionary neuroscience-based and intelligence-based explanations of the development of culture, for example, Holloway [20, 21]; Reader, Hager and Laland [22]; van Schick and Burkart [23]; Stout and Chaminade [24]; Whiten and van Schaik [25]. However, these researches have not offered detailed brain *mechanisms* that would offer (1) an explanation for how culture is uniquely learned through repetitive experience during socialization, or (2) an explanation of how such learning would differentiate uniquely human culture from complex group behavior among lower animals. For the reader who wishes more background on the evolution of culture, especially the evolution of how symbolic, linguistic, and cultural *capacities* might have emerged and developed in our species, Haidle, Bolus, Collard et al. [26] is recommended.

## Bringing in the other four-fifths of the neurons of the brain to more fully understand the evolution of culture

In their watershed articles, Leiner, Leiner and Dow [9, 10] pointed out that the human cerebellum increased three- to fourfold in last million years. They further pointed out that this huge increase in size of the cerebellum was linked by two-way nerve tracks (20 million on each side of the brain) to the cerebral cortex, including the parietal and prefrontal areas for planning and language functions (Leiner, Leiner & Dow [10]). Leiner, Leiner and Dow proposed that the evolutionarily differentiated development of the newer parts of the dentate nucleus of the cerebellum enabled the brain to unconsciously manipulate *ideas and their communication* with great dexterity just as the phylogenetically older portions of the dentate nucleus had done for motor skills. Today, such unconscious manipulation of ideas is referred to as unconscious processes in working memory [1, 27].

Leiner, Leiner and Dow’s [9, 10] foregoing early speculations and hypothesis concerning the cognitive functions of the cerebellum have been strongly supported by literally hundreds of brain-imaging and clinical studies. Among such studies particularly relevant to the present article are the following: Akshoomoff et al. [3]; Balsters, Whalen, Robertson et al. [28]; Ito [7, 8]; Leggio and Molinari [4]; Liao, Kronemer, Yau et al. [29]; Marvel and Desmond [30, 31]: Schmahmann [32]; Stoodley, Valera and Schmahmann [33]; Strick, Dum and Fiez [34]; Vandervert [1].

**Fig. 1:**
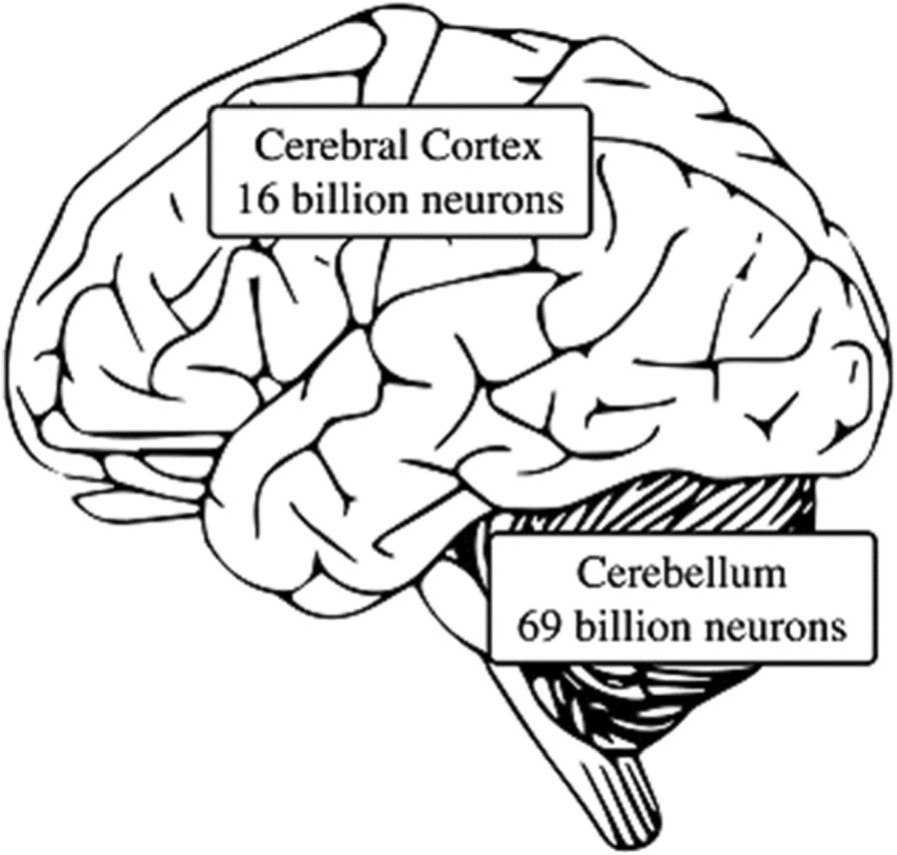
The number of neurons in the cerebellum versus the number in the cerebral cortex. Neuron count data from: Lent et.al. [35]

Figure 1 illustrates the enormous, 69 billion-neuron computational capacity of the cerebellum compared to 16 billion neurons in the cerebral cortex [35] that is proposed have been and continues to be behind the evolution of uniquely human culture. As Leiner, Leiner and Dow [9] argued three decades ago, critically important in this relationship has been the evolutionary expansion of the cerebellum’s dentate nucleus:

In the human brain, the dentate nucleus has become enormous, both when compared to other cerebellar nuclei and when compared with its size in other species…Its increase in size developed in parallel with the enlargement of the cerebral cortex and cerebellar cortex. (p. 444)

The dentate nucleus is composed of a phylogenetically older motor loop (dorsal dentate) and a cognitive loop (ventral dentate). In humans, the ventral dentate is twice as large as the dorsal dentate and is proportionately larger than that of the great apes (Bostan, Dum & Strick [36]).

Marvel and Desmond [30] suggested that the ventral dentate (cognitive loop) was naturally selected from the evolutionarily older dorsal dentate (motor) as the cerebellar cortex and frontal areas of cerebral cortex expanded over the last million years. The ventral dentate of the cerebellum outputs to the frontal and parietal areas of the cerebral cortex (working memory, executive functions including planning, and rule-based learning [7, 9, 34]. Through the dentate nucleus, then, the cerebellum is involved in the learning of countless internal models which are sent to the cerebral cortex for both motor and cognitive processing. Based on extensive research studies, Bostan, Dum and Strick [36] argued that the “signal from the dentate to the prefrontal and posterior parietal areas of the cortex [working memory, executive functions and rule-based learning] is as important to their function as the signal the nucleus sends to motor areas of the cerebral cortex” (p. 3). Thus, as a 69 billion neuron-strong computational system based on sequence detection and prediction (Leggio & Molinari [4]), the human cerebellum wields an “unconscious presence” in thought, behavior and affect that is commensurate with the immense learning requirements and apparently unlimited potential of the experience and products of culture.

## The collaborative roles of the cerebellum and the cerebral cortex in socialization toward the norms of culture

Doya [12] rigorously laid out the differences between the computational architectures of the cerebral cortex and the cerebellum. He argued that (1) the computational role of the cerebral cortex is context-dependent, essentially managing survival and maintenance operations in the conscious here-and-now context, and (2) the computational role of the cerebellum, on the other hand, is “context-independent,” which means, in accordance with Leggio and Molinari [4], the cerebellum learns internal models of control that by-pass the rigorous relearning of the requirements of here-and-now contexts by establishing feedforward models of behavior and thought to unconsciously predict, anticipate and deal with those repetitive situations. These context-independent computations produce forward-predicting cerebellar internal models which enable skillful, error-corrected manipulative control in everything from, for example, skillful sports performance to expert piano performance to the creative ideas and innovations that result from *repetitive* experience in science, religion, mathematics, art, music, daily routines and social relationships (Vandervert [1]; Vandervert, Schimpf & Liu [37]).

How, exactly, are we to understand Doya’s [12] analysis of these different but completely integrated roles of the cerebral cortex and the cerebellum? What does “context-independent” mean, how does it come about and to what does it apply? As described earlier in the Introduction section of this article, Akshoomoff, Courchesne and Townsend [3] and Leggio and Molinari [4] independently proposed that the cerebellum does indeed specialize in learning of unconscious, forward-predicting internal models that are sent to working memory and other sensory, motor and affective processes in the cerebral cortex. Akshoomoff, Courchesne and Townsend [3] described important detail on how the cerebellum builds a predictive, unconscious structure into its internal models:

The cerebellum is a master computational system that adjusts responsiveness in a variety of networks to obtain a prescribed goal [in Baddeley’s working memory model, this is the *attentional control* of the central executive] (Courchesne, 1995; Courchesne et al., 1994). These networks include those thought to be involved in declarative memory, working memory, attention, arousal, affect, language, speech, homeostasis, and sensory modulation as well as motor control. This may require the cerebellum to implement a succession of precisely timed and selected changes in the pattern or level of neural activity in these diverse networks [It would do this by learning internal models that would implement such changes.]. We hypothesized that the cerebellum does this by encoding (“learning”) temporally ordered sequences of multi-dimensional information about external and internal events (effector, sensory, affective, mental, autonomic), and, as similar sequences of external and internal events unfold, they elicit a readout of the full sequence in advance of the real-time events. This readout is sent to and alters, *in advance* [italics added], the state of each motor, sensory, autonomic, attentional, memory, or affective system which, according to the previous “learning” of this sequence, will soon be actively involved in the current real-time events. So, in contrast to conscious, longer time-scale anticipatory processes mediated by cerebral systems, output of the cerebellum provides moment-to-moment, unconscious, very short time-scale, anticipatory information. (pp. 592–593)

It is important to note here that *attentional control* as used in the context of this article refers to its learning within working memory in cerebellar internal models as described in the above quote by Akshoomoff, Courchesne and Townsend—see also Vandervert [1] in this regard.

Thus combining Doya’s [12] description of the differences between the computational architectures of the cerebral cortex and the cerebellum with Akshoomoff, Courchesne and Townsend’s above cerebellar sequence detection, “context-independent” refers to internal models learned in the cerebellum that replace the *original context* of *repetitious* learning required of any tasks, including difficult, time-consuming, higher-order cognitive tasks and task related to complex social interaction (the original “contexts”). For example, in relation to the original, often arduous context-dependent process of learning a complex musical piece, “context-independent” does *not* mean independent of the musical piece in question but rather independent of the original difficult, repetitive learning tasks that have been replaced by a template of internal models (neural circuits) in the cerebellum. In regard to such higher-order and social tasks, see particularly Doya ([12], p. 671); Akshoomoff, Courchesne & Townsend’s [3] above-quoted listing of declarative memory, working, attention, arousal, affect and so on; Leggio & Molinari’s [4] above-quoted listing of “inferences of higher-order processes,” and “other persons” as controlled objects. As an example of the learning of context-independent internal models for these highest levels of thinking, Ito [7] provided a straightforward discussion on how the cerebellum might learn internal models of complex mental models taking place in the parietolateral association area and then forwards them back to the cerebral cortex to carry out the original context-dependent task in an error-corrected and unconscious manner.

It is proposed that, through cerebrocerebellar collaboration, these context-independent cerebellar internal models provide the basis for both the unconscious learning *of* and ongoing participation *in* culture. The powerful *adaptive* value of such context-independent internal models in culture is twofold: (1) they enable humans to collectively think or perform faster, more appropriately and more consistently in a predictive, feedforward manner (Akshoomoff, Courchesne & Townsend [3]; Ito [7]; Leggio & Molinari [4]; Vandervert [1, 2], and (2) increasingly adaptive and increasingly complex mental models and movements connected to complex ongoing social and technological problems with a culture can be creatively developed though the *blending* of cerebellar internal models in the individual (Imamizu, Higuchi, Toda & Kawato [5]; Vandervert [1]; Vandervert, Schimpf & Liu [37]. Individuals then share the products of their new, creative blends of cerebellar internal models (new ideas, new technologies and so forth) with other members of culture, thus advancing culture as a whole. These new ideas and technologies in turn give rise to additional new creative blends of cerebellar internal, thus continually advancing culture (often rapidly in the manner of a positive feedback loop) to higher social, scientific, technological, and artistic levels. An example of the optimization of mental/manual skill (point 1 above) in many individuals throughout culture can be seen clearly in the process of learning cerebellar internal models through years of repetitive practice leading to errorless and seemingly effortless complex musical performance (Vandervert [1]). Recall, that “context-independent” does *not* mean independent of the musical piece in question, but rather independent of the original difficult, repetitive learning tasks that have been replaced by a template of internal models^3^. So, now, the pianist, for example, plays a complex concerto errorlessly without “thinking” about the original contexts of the practice sessions (see, for example, Parsons, Sergent, Hodges et al. [38]). The leading edge and heart of a culture is made up of many such highly-practiced individuals across all components of culture, religion, science, art, engineering, music, technology and so on. And, it is proposed that examples of the constant, cumulative advance of ever-new blends of internal models and thus the advance of culture (point 2 above) include the cumulative growth of agricultural methods, the relentless technological and artistic advances of ancient Egyptians, Greeks, Romans, and on into the likewise relentless technological and artistic advances of the modern world.

## The observational learning of cerebellar internal-models provides the “glue” for socialization toward the norms of culture

In the sharing of the above cumulative advance of new, creative blends of cerebellar internal models across individuals, observational learning is critically important. Observational learning is learning that occurs by observing the behavior of others. Observational learning is critically important during socialization today (see Endnotes), but was even more important among ancient peoples where the bulk of socialization occurred, not in schools with books and computers, but in everyday community and family activities (including often-repeated religious/political ceremonies and rituals) and in occupational apprenticeships. How are cerebellar internal models acquired through observational learning, and, specifically, how are they *accumulated* in a person-to-person manner that leads to advances in culture?

In this regard, Wolpert, Doya and Kawato [15] proposed that a high level of “control” and observational learning related to the nonverbal behavior and intentions of others can be based on cerebellar internal models of one’s own motor system:

We hypothesize that…during action observation the [one’s own] motor system can be used to understand the actions of others. This could be an efficient process because our CNS has learned to predict the consequences of actions on our own body [as a collection of controlled objects] and this can be used to make accurate predictions about others. (p. 597)

In this hypothesized scenario, the cerebellum is involved in a combination of a high level of “control” and observational learning of the behavior of another person. Recall from the discussion of “controlled objects” in the introduction of this article that in the above scenario the observed person would be a controlled object in the same sense that a person’s own arm is a controlled object manipulated by internal models in the cerebellum.

Along this same line of observational or imitative learning, cerebellum research has outlined detailed accounts of how internal models of communication between speakers and listeners and in the imitation of others operate in (1) advanced social interaction at the symbolic level (Wolpert, Doya & Kawato [15]), (2) higher-level, mutual mental modeling between speaker and listener during social interaction (Imamizu & Kawato [13]), and (3) the comprehension of sentences between speaker and listener (Moberget Gullsden, Andersson et al. [14]).

Following directly in this line of research, Doya [12] proposed that the cerebellum learns internal models for words and gestures between speaker and listener:

In the context of communication, the “environment” is the partner [the listener] of communication and the goal is to bring the physical or internal state of the partner into a desired state. This involves sequential selection of actions, i.e. words or *gestures* [italics added], in an appropriate sequence, in the same way as in the case of many control tasks. When the model of the partner is available [for example, a close friend or a familiar teacher], the goal can be achieved more readily and quickly. If the internal models of the *speaker* [italics added] and *listener* [italics added] are similar, communication [and, thus observational learning] is made efficient. (pp. 970–971)

While not all observational learning includes such speaker/listener modeling, most family, school, religious learning, apprentice situations and so forth occurring during socialization into the norms of culture do. It is important to note in this regard, that in situations where there is a good degree of one-sided or one-way communication (for example, teachers presenting lessons to students without interaction, children watching television or listening to parent conversations, and so forth) the listener would experience a diminished degree of the learning of forward-predictive cerebellar internal models of the “speaker.” This is so, because as compared to two-way communication, these one-way situations permit cerebellar internal models to test and error-correct diminished samples of the speaker’s (or television program’s) behavior and mental state(s).

It is proposed that it is cerebellar internal models, learned largely through observational learning and in accordance with Leggio and Molinari’s [4] earlier above-quoted sequence detection process that provide the substance and “glue” that binds individuals together in both the experience and capacity to participate in a common culture. Without the concepts initiated by cerebellar internal models through repetitious learning of common tasks in each individual’s brain, there would be no conscious/unconscious common framework to bind the members of culture together in a “silent” fashion, again, for example, in art, science, religion, music, mathematics, politics, child-rearing and so on. The expression that members of culture are bound together in a “silent” fashion is used here to draw attention to this important point that Leiner, Leiner and Dow [10] made on the unconscious nature of learning in the cerebellum:

Cerebellar signals are always generated below the level of conscious awareness in the brain. How cerebellar contributions can improve the speed and skill of cerebral performance is therefore not accessible to conscious introspection. Rather, the cerebellar contributions to cognitive and language skills would constitute a part of what is called ‘the cognitive unconscious.’ (p. 1006)

## Important implications of the prominent role of the cerebellum in the learning of culture

In summary to this point, it is proposed that the learning of the components of culture can best be understood and studied as the learning of a general template of cerebellar internal models. This template of cerebellar internal models unconsciously controls the focus, shifting, and duration of attention in working memory, affect and so forth as it is shared as “moment-to-moment, unconscious, very short time-scale, anticipatory information” (Akshoomoff, Courchesne, Townsend ([3], p. 593), see their earlier above quote) among the members of culture. Following Baddeley [39], these parameters of attention provide executive control for ongoing thought including constant, ongoing access to cultural information held in long-term memory.

## Cultural deprivation and cultural shifts, and the cerebellar cognitive affective syndrome

This view of the cerebellum’s role in the learning of culture offers a way to study the effects of cultural practices, cultural changes, and importantly, cultural deprivation and maltreatment on the development of working memory, and, as Ito [7] described, how those changes might affect both normal and abnormal mental and affective development:

In analogy to the contribution of the cerebellum to motor activity, its contribution to mental activity may be specified as regulating the speed, consistency, and appropriateness of cognitive processes, with dysfunction leading to a dysmetria of thought (Schmahmann [40]). This provides theoretical bases for explaining cerebellar symptoms such as dysmetria as being due to impairment of a cerebellar model of musculoskeletal system. A similar explanation applies to *mental dysmetria* [italics added] that may occur due to lack of the [cerebellar internal] model which copies a mental model [of the cerebral cortex]. (p. 486)

Specifically, Schmahmann [18] proposed, that such dysfunction of the cerebrocerebellar circuits/functions produces dysmetria of thought with the following mental and affective characteristics:

It [the cerebellar cognitive affective syndrome] is characterized by (1) disturbances of executive function, which includes deficient planning, set-shifting, abstract reasoning, working memory, and decreased verbal fluency; (2) impaired spatial cognition, including visual-spatial disorganization and impaired visual-spatial memory; (3) personality change characterized by *flattening or blunting of affect and disinhibited or inappropriate behavior*; and (4) linguistic difficulties, including dysprosodia, agrammatism and mild anomia. *The net effect of these disturbances in cognitive functioning was a general lowering of overall intellectual function* [italics added]. (Schmahmann [18], p. 371

It is proposed that these functions are importantly related to the early development of mental and affective components of communication (language and nonverbal communication) (a la Doya’s [12] above quote) by which, to a large degree, culture is transmitted. This idea is supported by Knickmeyer, Gouttard, Kang, Evans, Wilber, Smith et al. [41] who argued that the 240 % increase in the size of the cerebellum in the first year suggested the great sensitivity of the cerebellum to experience during the first year and on into the school years:

Because the cerebellum is critically involved in motor coordination and balance [42] the striking cerebellar growth may underpin the rapid motor developments of infancy. The cerebellum has also been implicated in a plethora of other cognitive abilities including planning, set-shifting, language abilities, abstract reasoning, *working memory* [italics added], and *visual-spatial organization* [italics added] [17]. Given that “cognitive” regions of the cerebellum have reciprocal projections with nonprimary frontal, parietal, and occipital association cortex [43], the extremely rapid growth of the cerebellum in the first year may be a prerequisite for specific aspects of later cortical development. ([41], p. 12180)

It is therefore suggested that through a number of types and degrees of cultural deprivation the functions listed in Schmahmann’s above cerebellar cognitive affective syndrome may be impaired during socialization/enculturation (see Endnotes).

## Cultural deprivation impairs the acquisition of social cues

Studies on the effects of socialization on the development of the cerebellum have offered preliminary support for this suggestion. For example, Bauer, Hanson, Pierson et al. [44] studied the effects of cultural deprivation on the development of the cerebellum and cognitive functions of young children in austere orphanages^4^. This research found that orphanage-induced social/cultural deprivation resulted in significantly smaller left and right superior-posterior cerebellar lobe volumes and, cognitively, reduced visual-spatial memory and reduced attentional and planning components of executive function. In addition, Baldacara, Jackowski, Schoedl et al. [45] also found that emotional maltreatment during such institution-induced socialization leads to reduced cerebellar volume and to various degrees of the “flattening or blunting of affect and disinhibited or inappropriate behavior” cited among the symptoms of dysmetria of thought by Schmahmann [18] above.

Corroborative evidence that the cerebellum is the key driver of socialization and, thereby, enculturation also comes from an unexpected source. Giedd [46] pointed out that electronic media (television, cellphones, the Internet, etc.) have changed the way children and adolescents learn, play, and socialize more in the last 15 years than in the previous 570 some years since the introduction of Gutenberg’s printing press. Because the increase in the use of these media (mostly television, see Rideout, Foehr and Roberts [47]) have dramatically changed learning, play, and socialization among children, it represents a definite and significant cultural modification or “shift.” The actual existence of this suggested cultural shift is strongly supported by a preponderance of findings that, much like Bauer et al.’s [44] and Baldacara et al.’s [45] above-cited social/cultural deprivation-induced effects, excessive television viewing among children produces what might be considered mild, parallel appearances of Schmahmann’s [18] cerebellar cognitive affective syndrome. For example, studies on both excessive and specialized aspects of television viewing have found that those same (but less pronounced) negative attentional, executive, and affective effects occur (Lillard & Peterson [48, 49]; Pagani, Fitzpatrick & Barnett [50]; Watt, Fitzpatrick, Derevensky et al. [51]). As Bauer et al. [44] and Baldacara et al. [45] found for institutional deprivation, these researchers suggested that excessive television viewing deprives young children of critical developmental socialization and peer-interactive play activities.

## Learning to be a bystander: excessive television viewing reduces the Cerebellum’s learning of other-persons-as-control-objects

All of the foregoing research concludes that the negative effects of both institutional deprivation and excessive television viewing are the result of reduced opportunities for hands-on socialization. But how, exactly, does this reduced socialization occur in the brain? Within the framework of cerebellar internal models described in this article, it is suggested that these negative socialization effects are not only the result of the learning of a lessened degree of socialization but also the result of the learning of internal models for *a different kind of socialization*. Although this suggestion of a different kind of socialization can apply in either institutional deprivation or excessive television viewing, an example will be given only for the case of excessive television viewing. Again returning to Doya’s [12] and Wolpert et al’s [15] above notion of speaker and listener-as-control-objects in interpersonal language and nonverbal communication, it is proposed that when a child watches characters in scenarios on television (either cartoon characters or actual persons) they are still learning cerebellar internal models related to “socialization,” but with increased television viewing it is increasingly a *one-sided* or “bystander” template of internal models that is being learned rather than one of a socially richer two-sided interaction. This television-mediated, one-sided socialization is less demanding of the unconscious learning of cerebellar control models (for example, requires less hands-on social give-and-take communication and eye-contact) in the control of attention, executive control, and affect as described by Akshoomoff, et al. [3]. This occurs in television viewing it is suggested, because the other “person(s)” are either more predictable because television plots are very similar, or their behavior and thoughts need not be predicted at all because there are no real-world consequences if the television viewer does not learn to predict them (see Leggio & Molinari’s [4] quote on cerebellar predictive sequence detection above). In fact, the other persons or cartoon characters seen in television programming may be more entertaining, because they are *not* predictable, or prediction is elusive^5^.

In essence, today’s children in varying degrees are learning to be a part of a “media-mediated” culture that is quite different from that of previous generations. They are learning to become a part of the rapidly emerging electronic media culture described above by Giedd [46]. As Giedd suggested, some of the effects of what in this article is called one-sided socialization are positive (for example, in opening new horizons of information access) and some are negative (as in lowering attentional control, school grades, and social adjustment as found by Lillard et al. [48, 49], Pagani et al. [50] and Watt et al. [51]. It is proposed that the negative effects are the result of mild impairment of the development of *implicit* learning via cerebellar internal models as described by Ito [8], leading to an equally mild, parallel development of Schmahmann’s [18] cerebellar cognitive affective syndrome (see component characteristics listed earlier above). This contention is supported by D’Mello and Stoodley’s [52] in comments on the role of cerebellar internal model in implicit learning, especially in early development:

Abnormal connectivity between the cerebellum and cerebral motor regions might result in sub-optimal automatization and modulation of motor behaviors, and might also be related to delayed acquisition of gestures important for social interaction and communication. Similarly, abnormal connectivity between the cerebellum and cerebral cortical regions involved in language could lead to atypical organization of language networks in ASD (autism spectrum disorder), and be associated with delayed language acquisition in ASD. (p. 13)

## Conclusions and discussion

It is concluded that the essential components of culture are learned and sustained not by the cerebral cortex alone as many traditionally believe, but are substantially learned in cerebellar internal models through repetitious experience. Following Akshoomoff, et al. [3]; Imamizu, Higuchi, Toda & Kawato [5]; Ito [7, 8, 53, 54] and Leggio & Molinari [4] these cerebellar internal models were adaptive because, (‘1) by encoding (“learning”) temporally ordered sequences of multi-dimensional information about external and internal events, they predicted future events in advance, (2) through constant error-correction, they regulated the speed, consistency, and appropriateness of movement and thought in the cerebral cortex, and (3) when confronting new tasks, they are blended to provide new solutions. It is further concluded that, in the process of socialization (see Endnotes), these cerebellar internal models are largely derived through observational learning from communication (including gestures) shared among members of a particular group of people a la Doya, [12], Imamizu & Kawato, [13], Moberget et al. [14], and Wolpert et al. [15]. These internal models are generated below the level of conscious awareness (Leiner, Leiner & Dow [10]), and, it is suggested, are responsible for predicting behavioral and cognitive requirements necessary in the origin of culture, for the participation in culture, and the forward advance of culture.

The cerebellar approach to the nature and origins of culture offers the following new explanations for a number of important questions.

*First*, by recognizing that all people learn similar cerebellar internal models to similar repeated acts and experiences (Ito [7]; Leggio & Molinari [4]), the cerebellar approach explains how the components of culture, although observed in others and taught by others, can be *unconsciously* learned by each person, *but yet be learned to be in close sync with others of the same group*. That is, the unconscious learning of cerebellar internal models through speaker-listener communication (including nonverbal communication [Doya [12]) reduces a myriad of similar environmental/social circumstances to predictive, error-adjusted (Leggio and Molinari [4]) social principles which drive social thought, behavior and affect in a similar manner in all members of the social group.

*Second*, the larger anthropological context for the evolution of the origin of human culture now appears to have been the adaptive natural selection of sequence-based (rule-based) cognitive processes required in the natural selection of the manufacture and use of stone tools beginning some one and a half million years ago (e.g., Barton & Venditti, [55]; Greenfield, [56]; Leiner, Leiner & Dow, [9, 10]; Stout & Chaminade, [24]; Vandervert, [1, 11, 57, 58]). Because of its requirement of prolonged cognitive effort, this adaptive selection advantage of stone-tool manufacture and use likely selected toward the three- to fourfold expansion of the size of the cerebellum and, and especially its cognitive, working memory functions, which Leiner et al. [9] referred to as “the skillful manipulation of ideas” (p. 444). Within this context of adaptively evolving cerebrocerebellar feedback loops and the slowly accelerating complexity of stone-tool production (Ambrose, [59]), it is proposed that *the earliest shared, highly-repetitive, sequential motor/cognitive activity* necessary for a cerebellum-driven “culture” would have likely developed. This offers a cerebellum internal models-based explanation for how culture could have originated out of mutually-shared observational learning related to the tool-manufacturing and tool-using other persons-as-control-objects (Doya, [12]). It is suggested that, within this highly-repetitive, sequential activity and based on the blending of cerebellar internal models (Imamizu et al. [5]) was the beginning of a *positive feedback loop* (what was learned and produced in turn led to greater, more refined learning and production). This scenario compares directly with anthropologist Ralph Holloway’s brain-culture positive feedback loop ([20], pp. 293–295).

*Third*, Vandervert [1, 2, 57] and Vandervert, Schimpf and Liu [37] described an evolutionary scenario that (1) cerebellar internal models are blended in the process of optimizing problem solving in working memory (Imamizu et al. [5]; Ito, [49]; Yomogida, Sugiura, Watanabe et al. [60]), and (2) that when these newly blended internal models are sent to consciousness in the prefrontal executive and parietolateral association cortices (working memory areas) (Ito, [53]), they may be experienced as sudden, intuitive new solutions to problems. Within the larger context of the first point above, this offers an explanation not only for individual creativity but the constant creative, forward advance of culture as a whole (Vandervert, [2, 57]; Vandervert, Schimpf & Liu [37]).

*Fourth*, Vandervert [11] proposed that within the context of gradually more adaptive manufacture and use of stone tools (especially the last million years) that cerebellar internal models adaptively *blended* (Imamizu et al. [5]; Yomogida et al. [60]) visual-spatial working memory with vocalizations to produce symbolic, syntactical language^6^. According to Vandervert [11], this latter adaptation was the basis of the evolution of Baddeley’s phonological loop from the existing, pre-language visual-spatial working memory in earlier Homo erectus. It is suggested that the evolution of this new symbolic level of communication produced more readily communicated (a la Doya [12]) details of ongoing socially shared experience. This idea is strongly supported by Van Overwalle and Marien's [16] conclusion that the cerebellum learns internal models for moment-to-moment, predictive “fluent and automatic social interaction” (p. 16). Vandervert [11] suggested that the foregoing cerebro-cerebellar blending of (1) a sequence-driven, decomposed visual-spatial experience with (2) vocalizations likewise selectively decomposed toward language led to a highly adaptive, infinitely partitionable^7^ internal model input (language) into working memory. That is, the gradual emergence of an *infinitely partitionable* working memory, and, at the same time, a socially sharable working memory, a la Van Overwalle and Marien's above “fluent and automatic social interaction,” would have been an enormous selective advantage. It is suggested that this adaptive selection, shared through the emerging phonological loop a la Doya [12], offers an explanation of the adaptive beginning of culture as a sharable, *infinitely partitionable reality* within working memory. It seems only sequence-detecting, error-driven cerebellar internal models (Leggio & Molinari, [4]), in collaboration with the advance human cerebral cortex, had uniquely evolved to produce such an outcome.

*Fifth*, the cerebrocerebellar approach to the origin and nature of culture described herein offers a brain-based explanation of how excessive television viewing (a profound cultural shift which has occurred Giedd, [46]; Rideout, Foehr and Roberts [47]) especially among children, might disrupt traditional, pre-television, two-sided socialization which Lillard et al. [48], Pagnani et al. [50] and Watt et al. [51] found reduces attentional control, school grades, and social adjustment. This explanation proposes that excessive television viewing diminishes social interaction with other-persons-as-cerebellar-control-objects (a la Doya, [12]; Imamizu & Kawato, [13]; Moberget et al. [14]; Wolpert et al. [15]) and replaces “other persons” with non-interactive (and therefore inconsequential) “persons,” *and thereby reduces the repetitious or implicit aspect of observational learning.* It is suggested that this results in the learning of cerebellar internal models for a one-sided socialization that is similar in effect to that of socially abused children raised in socially austere orphanages (non-interactive caretakers, little play) found by Bauer et al. [44]. As D’Mello and Stoodley [52] suggested for implicit motor and cognitive learning especially during the early developmental years, it is further proposed, that this one-sided, unconscious learning of cerebellar internal models results in diminished learning of attentional, executive, and affective functions, in other words, a mild, parallel learned version of Schmahmann’s [18] cerebellar cognitive affective syndrome.

In summary it is concluded that culture is a collaborative outcome of the cerebral cortex and the cerebellum. It is hypothesized that the cerebellum, through the evolutionary differentiation of its dentate nucleus toward cognitive functions including working memory and language [9, 10, 30, 36], plays the more prominent role in the learning, maintenance, and advance of culture. It is suggested that Ito’s [8] conception of the “implicit self” (learned through repetition below the level of conscious awareness) is embedded within the proposed largely cerebellum-driven model of culture.

## Footnotes

1 Culture is defined [here] as the shared patterns of behaviors and interactions, cognitive constructs, and affective understandings that are learned through a process of *socialization* [italics added]. These shared patterns identify the members of a culture group while also distinguishing those of another group. Source: Center for Advanced Research on Language Acquisition (http://www.carla.umn.edu/culture/definitions.html). Socialization is defined by Sensoy and DiAngelo [61] as follows: “Socialization refers to our systematic training into the norms of our culture. Socialization is the process of learning the meanings and practices that enable us to make sense of and behave appropriately in that culture” (p. 15).

2 The computational role of the cerebellum is to learn “context-independent” internal models (Doya [12]), which means it learns internal models of control that by-pass the rigorous, constant relearning of the requirements of repetitive here-and-now contexts of the cerebral cortex. It does this by unconsciously learning feedforward models of behavior and thought to predict, anticipate and, though error-correction, optimally deal with those repetitive situations (Leggio & Molinari [4]).

3 Music training, by the very nature of its cognitive, affective and manual production requirements, draws heavily on the components and capacities of working memory. Broadly, working memory is the capacity to temporarily maintain and subsequently manipulate information “online” in the pursuit of goal-oriented tasks (see, for example, Baddeley [62]). In music training for the piano, for example, these working memory tasks include reading the musical notation system and sustaining the highly attentive repetitious practice required to learn the transfer of this notation into sequences of bimanual production, the latter of which depends on multisensory feedback. Through practice these rigorous context-dependent requirements are by-passed by the learning of cerebellar “context-independent” internal models.

4 The level of social austerity in orphanage institutions varies greatly. However, the institutions in these studies were generally characterized by unfavorable child-to-caregiver ratios, regimented daily living routines, and diminished sensory, cognitive, and language stimulation, and diminished social responsiveness of caregivers.

5 Learning internal models of other-persons (or cartoon characters)-as-control-objects that *don’t predict* future behavioral or mental requirements may seem to contradict Akshoomoff et al’s [3] and Leggio and Molinari’s [4] proposals that the cerebellum’s mode operation is to *predict*. However, all functions of working memory are aimed at and honed (by the repetitive learning of error-correcting cerebellar internal models) toward the accomplishment of a prescribed goal (Cowan, [63]; Fuster, [64]; Miyake & Shaw, [65]) formulated, not just in the cerebellum, but in the history of collaboration of the cerebral cortex and the cerebellum. This includes imaginary scenarios as goals (for example, Yomogida et al. [60]). Therefore, a prospective goal in working memory may be to achieve pure fantasy in science fiction novels, television shows or cartoons (where common sense and the laws of physics are humorously or interestingly violated). In the case of such fantasy in working memory, cerebellar internal models are honed toward the prospective goals of the imagined “logic” of the story.

6 This evolutionary blending of visual-spatial working memory with vocalization is generally supported by Liao, Kronemer, Yau, Desmond and Marvel [29] who demonstrated that the motor cortex and superior cerebellum are involved in the rehearsal/maintenance of both verbal and nonverbal (pictorial) content in working memory. Liao et al. may reasonably be interpreted to reveal how the original evolution of the adaptive blending of visual-spatial-related motor and vocal-motor processes remains an equally adaptive mechanism for the reinforcement of memory traces during verbal working memory.

7 Baddeley [62] suggested a similar evolutionary scenario for working memory: Working memory stands at the crossroads between memory, attention, and perception. In the case of the slave systems, the phonological loop, for example, probably represents an evolution of the basic speech perception and production systems to the point at which they can be used for active memory. (p. 559). Directly following and supporting Baddeley’s scenario, Vandervert [11] argued that the vocal tagging and filing in long-term memory during the evolutionary emergence of the phonological loop was the result of the following two interrelated contributions of cerebro-cerebellar collaboration. First, upon encountering new, challenging environmental demands which pressed the limits of then-existing stone tool technology, cerebellar internal models gradually decomposed/re-composed visual-spatial experience associated with situation-specific actions, and parallel situation-specific vocalizations into further decompositions/re-organizations of cerebellar internal models (Flanagan et al. [66]; Haruno, Wolpert, and Kawato, [67]; Nakano et al. [68]) which, when blended, selected toward new, uniquely human syntactic orders of language features. Second, these new vocal differentiations in evolving visual-spatial working memory served as an increasingly larger system of associative internal and social vocal tags (Fuster [64], pp. 249–251).
